# Impact of Iodinated Contrast Media in Patients Received Percutaneous Coronary Intervention: Focus on Thyroid Disease

**DOI:** 10.3389/fendo.2022.917498

**Published:** 2022-06-23

**Authors:** Yasha Chen, Xueyang Zheng, Na Li, Wenhao Niu, Bowen Hu, Xun Yuan, Chun Liang, Yunling Lin

**Affiliations:** ^1^ Department of Cardiology, Shanghai Changzheng Hospital, Navy Medical University, Shanghai, China; ^2^ Department of Cardiology, Fujian Medical University Union Hospital, Fujian Institute of Coronary Artery Disease, Fuzhou, China; ^3^ Department of Organ Transplantation, Shanghai Changzheng Hospital, Navy Medical University, Shanghai, China

**Keywords:** iodinated contrast media, coronary artery disease, percutaneous coronary intervention, thyroid disease, hyperthyroidism

## Abstract

**Background:**

With the rapid advance in percutaneous coronary intervention (PCI) technology, patients absorb large volume of iodinated contrast media (ICM). Recent studies suggested that ICM may lead to hyperthyroidism, but the association between ICM volume and thyroid is still unclear. We sought to evaluate the long-term influence of ICM on thyroid dysfunction and disease in patients received PCI.

**Methods:**

This single-center retrospective study included consecutive coronary artery disease (CAD) patients. A covariance (ANCOVA) model was performed to evaluate the change of serum TSH, FT3 and FT4 before and one-year after the PCI procedure. Restricted cubic splines and logistic regression were performed to evaluate the association between ICM volume and thyroid disease.

**Results:**

2062 patients met inclusion criteria (1381 patients in the low-volume group and 681 patients in the high-volume group). The high-volume group was 0.238 ± 0.092 pmol/L higher than the low-volume group (*P* = 0.010) in the serum FT4. Restricted cubic splines show that there were linear dose-response relationships for ICM volume and composite endpoint and hyperthyroidism. In all models, there were significant differences in composite endpoint between the two groups. (*OR* 1.75, 95% *CI* (1.05, 2.92), *P* = 0.032, *OR* 1.73, 95% *CI* (1.01-2.96), *P*= 0.032 and *OR* 1.83, 95% *CI* (1.09-3.06), *P*= 0.022, respectively). The positive results were also showed for hyperthyroidism in all models (*OR* 2.35, 95% *CI* (1.14-4.84), *P* = 0.021, *OR* 10.36, 95% *CI* (1.20-89.00), *P* = 0.033 and *OR* 2.35, 95% CI (1.13-4.87), *P* = 0.022, respectively).

**Conclusion:**

The present analysis gives an overview that ICM volume is associated with an increased risk of thyroid dysfunction and thyroid disease.

## Introduction

The practice of coronary angiography and percutaneous coronary intervention (PCI) procedures has heavily relied on iodinated contrast media (ICM). The rapid advance in PCI technology has greatly improved the diagnosis and treatment for consecutive coronary artery disease (CAD) patients. Unfortunately, because of the aging population and burden of complications, the overall quantity and complexity of PCI have increased (1). In a routine PCI procedure, the patient may absorb around 200 times the daily requirement for iodine. The nephrotoxicity effect of ICM has been widely and deeply explored (2, 3). However, a few studies have evaluated the effects of ICM on the normal thyroid gland of patients received PCI (4–7), and no well-defined protocols exist to guide the administration for this population.

The thyroid gland utilizes free iodide in serum to synthesize thyroid hormone for metabolic functions. The normal thyroid gland possesses the ability to adapt to the increasement of iodine. But rapid increase of serum iodine may prevent normal adaptation and either hypothyroidism or hyperthyroidism may occur. Overload iodine can lead to suppression of synthesis of thyroid hormone (Wolff-Chaikoff effect) (8). Iodine may also inhibit thyroid hormone release and either of these phenomena can lead to the development of hypothyroidism. Inversely, high iodine levels may cause hyperthyroidism (or Jod-Basedow disease) (9). Worse, the prolonged hyperthyroid or hypothyroid status may pose negative effects on cardiovascular disease and survival (10).

Until now, the risk of ICM has not been clarified in patients with euthyroidism received PCI. Hence, we conducted this retrospective cohort study to determine whether ICM exposure is associated with thyroid dysfunction and thyroid disease in the PCI population.

## Materials and Methods

### Study Design and Population

**Figure 1:**
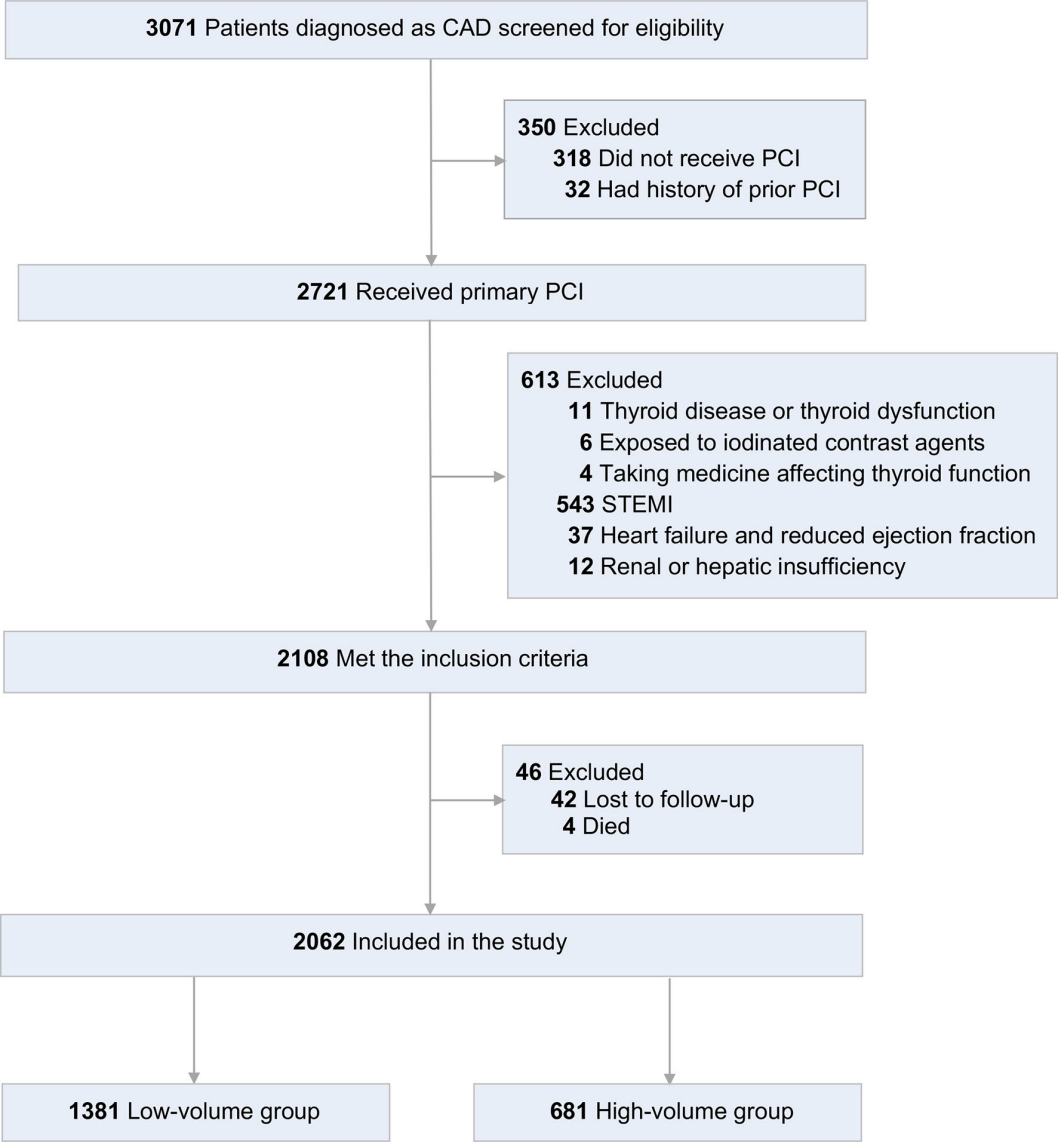
Flow diagram for identifying study population. CAD, coronary artery disease; PCI, percutaneous coronary intervention; STEMI, ST-segment elevation myocardial infarction.

This retrospectively designed observational study included all consecutive patients who were diagnosed with CAD from January 2015 to December 2017 in Fujian Medical University Union Hospital. According to the American College of Cardiology/American Heart Association 2014 criteria, CAD was defined as follows: defined as ≥ 50% vessel stenosis or having undergone interventional therapy according to coronary angiography results (11). Inclusion criteria were: (A) patients with age equal to or over 18 years; (B) patients received primary PCI as a management strategy; (C) patients completed an one-year follow-up. Exclusion criteria were: (A) were suffering or had suffered thyroid or metabolic disease or thyroid dysfunction; (B) were suffering or had suffered hypothalamic or pituitary diseases; (C) had exposed to ICM, including prior PCI; (D) were with a current prescription for levothyroxine, antithyroid drugs, amiodarone or lithium; (E) were STEMI (ST-segment elevation myocardial infarction); (F) with heart failure or reduced ejection fraction (< 50%); (G) with hepatic or renal dysfunction. Eligible patients were divided into two groups according to volume of ICM: low-volume group (≤ 200ml) and high-volume group (> 200ml). The flow diagram for identifying the study population was shown in **Figure 1**.

The study was conducted in accordance with the Declaration of Helsinki (as revised in 2013) and approved by the ethics committee of Fujian Medical University Union Hospital (No. 2019JYKY010). According to local policy, informed consent was waived due to the retrospective nature of the study.

### Treatment Strategy and Data Management

All data was extracted from the hospital’s electronic database. The following demographic, clinical and laboratory data were exported from the medical hospital electronic database within one day of admission independently by 2 investigators: demographic characteristics (age, gender, weight and height at admission), behavioral habit (current smoker), medical history (hypertension, diabetes, hyperlipidemia, peripheral vascular disease and stroke), clinical diagnosis (unstable angina pectoris, stable angina pectoris and non-ST-segment elevation myocardial (NSTEMI), PCI procedure characteristics (number of implanted stents and ICM volume) and medications at baseline (aspirin, clopidogrel or ticagrelor and statins). Patients were asked to bring all their medication to the interview before the start of treatment.

Thyroid function tests were conducted in all patients 24 to 48 hours before coronary angiography. Serum TSH, free triiodothyronine (FT3) and free thyroxine (FT4) were measured at the Fujian Medical University Union Hospital Immuno-Serology Laboratory. Blood samples were obtained during fasting. Serum was separated and stored at −80°C till the end of the study for measurement of thyroid hormones. TSH was measured using the immune enzymatic Access TSH (3rd IS) assay (Beckman Coulter, Brea, CA) in a DXI 800 analyzer (Beckman Coulter); TSH reference range: 0.27-4.20 mIU/L. FT3 and FT4 were measured using the FT3 and FT4 ADVIA Centaur immunoassay (Siemens Healthcare Diagnostics) in an ADVIA Centaur analyzer (Siemens Healthcare Diagnostics, Tarrytown, NY); FT3 reference range: 3.10-4.80 pmol/L. FT4 reference range: 12.00-22.00 pmol/L. Both the laboratory and the specific assays are certified for clinical use.

Patient treatment was performed according to the current standard practice of coronary heart disease by qualified cardiologists (12, 13). All patients were required to take aspirin plus clopidogrel or potent P2Y12 inhibitor for 1 year in the absence of contraindications. Coronary angiography was performed using the Judkins technique by two experienced interventional cardiologists. Significant coronary artery stenosis was diagnosed visually if accumulating plaque has narrowed diameter of coronary arteries equal to or over 50%. Complex PCI was defined as any of the following: 3 vessels treated, equal to or over 3 lesions treated, total stent length over 60 mm, bifurcation with 2 stents implanted, atherectomy device use, left main PCI, surgical bypass graft or chronic total occlusion as target lesions (14–17). All patients underwent coronary stent implantation with pre-dilatation by balloon. The type of implant-stent and intervention strategy was determined by interventionists.

Technical success was defined as the achievement of less than 20% final diameter stenosis after stent implantation and less than 50% after coronary angioplasty without stent implantation in visual assessment associated with Thrombolysis In Myocardial Infarction (TIMI) flow grade equal to or more than 3 (18). Procedure details including time of contrast administration, contrast volume, contrast administration route was recorded during the procedure. The contrast volume was measured in a 1-mL increment, with the total established from the manual injection used during the procedure. All procedures were done with the same injector type (Angiodyn angiographic syringes for manual contrast medium injection). The contrast volume was measured precisely with manual iodinated contrast delivery. All patients received Iohexol (Omnipaque, 300 mg iodine/mL; GE Healthcare, Chicago, IL, USA), a low osmolar and nonionic contrast medium, for coronary angiography and PCI.

### Study Visits and Follow-Up

Face-to-face clinically follow-up was scheduled on the 1^st^ month, 3^rd^ month, 6^th^ month and 12^th^ month after PCI. Visit windows are ±3 days for Month 1, ± 7 days for Month 3 and 6, and ±10 days for Month 12. At each study visit, thyroid function including TSH, T3 and T4 were measured and thyroid dysfunction-related symptom, including palpitations, weight loss, tremor, insomnia, anxiety, diarrhea, fatigue, cold intolerance, constipation, weight gain, delayed relaxation of deep tendon reflexes, bradycardia, were evaluated. If patients were suspected of thyroid related disease according to above evaluation, they would be further diagnosed with thyroid ultrasound and treated in the endocrinology department.

### Study Endpoints and Definitions

This study was powered for a 1-year endpoint: thyroid disease. The composite endpoint was a combination of overt hyperthyroidism, subclinical hyperthyroidism, overt hypothyroidism and subclinical hypothyroidism. Overt hyperthyroidism was defined as low serum TSH concentrations and raised serum concentrations of thyroid hormones: FT_4_, FT_3_, or both (19). Subclinical hyperthyroidism was defined as a subnormal serum TSH level along with serum FT4 and FT3 concentrations within the normal reference ranges (20). Hyperthyroidism included overt hyperthyroidism and subclinical hyperthyroidism. Overt hypothyroidism was defined as TSH concentrations above the reference range and free thyroxine concentrations below the reference range (21). Subclinical hypothyroidism was defined as an elevated serum thyrotropin level and a serum-free thyroxine level within the reference range (20). Hypothyroidism included overt hypothyroidism and subclinical hypothyroidism.

### Statistical Analysis

Before further analysis, categorical variables were expressed as a percentage (number) and analyzed by the chi-square test or Fisher exact test. For continuous variables, the mean or median in each group, depending on the distribution, was calculated with measures of dispersion (*SD* or *IQR*). Normally distributed variables were compared using independent sample t test, and non-normally distributed variables using the Mann-Whitney test. Counting variables were compared using the chi-squared test. For the evaluation of change in serum TSH, FT3 and FT4, parallelism test was conducted to make sure the homogeneity variances were compared. And between-group comparisons were performed using an analysis of covariance (ANCOVA) model adjusted for baseline value with baseline, group, and baseline by group interaction as factors. Restricted cubic splines (RCS) were applied to visualize the dose-response association between ICM volume and clinical endpoint based on odds ratios with logistic regression.

Binary logistic regression analysis using enter method was performed to assess the endpoints between two groups. The initial model was adjusted for age, gender and obesity. The second model was additionally adjusted for the current smoker, hypertension, hyperlipemia and previous stroke based on Model 1. Results were expressed as *OR* (*OR* less than 1.0 favored low-volume group, *OR* equal to or over 1.0 favored high-volume group) with 95% *CI*. The goodness of fit was evaluated using the Hosmer-Lemeshow test. Further analyses were performed based on the non-complex PCI group and complex PCI group for the 1-year clinical endpoints. All probability values were 2-tailed, and a *P* value less than 0.05 was considered statistically significant. The statistical analyses were done with software IBM SPSS. 23.0 (SPSS Inc, Chicago, IL, USA) and R (version 3.6.3).

## Results

### Study Procedure and Baseline Characteristics

From January 2015 to December 2017, 3071 patients with CAD were assessed for eligibility. 350 patients were excluded for did not receive PCI or had a history of prior PCI. Of the 2721 patients received primary PCI, 2108 patients met the inclusion criteria. Then, for lost to follow-up or died, 46 patients were excluded. Finally, 2062 patients were included in the study (**Figure 1**). Patients had a mean age of 62.28 ± 9.8 years; 81.0% were men; 42.4% were current smokers; 42.8% had a history of hypertension, 31.4% diabetes, 35.5% hyperlipidemia, and 9.7% stroke.

### ICM Volume and Thyroid Disease

**Figure 2:**
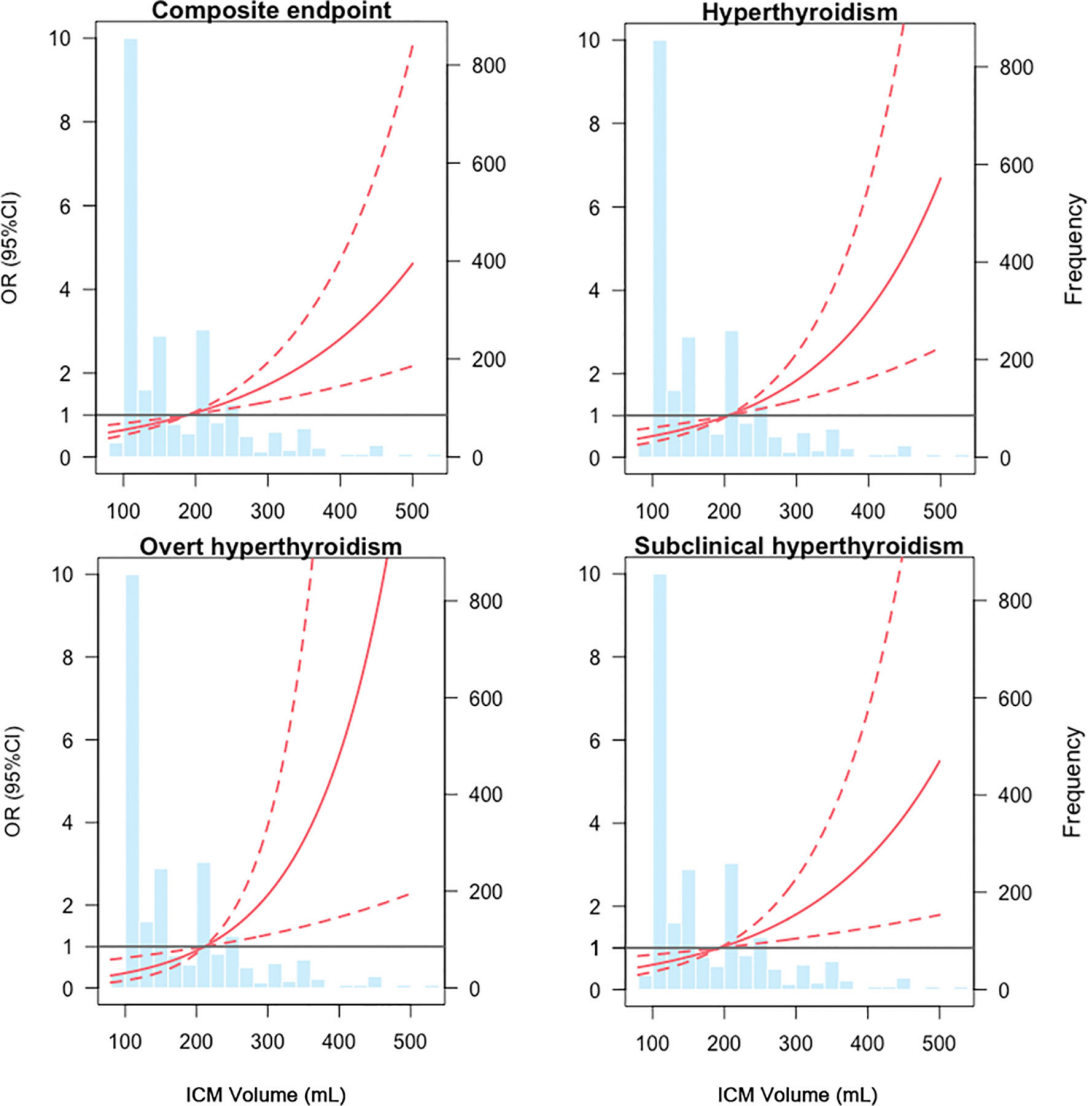
Dose-response relationships between ICM volume and composite endpoints from restricted cubic splines model. The solid red lines are the estimate of odds ratios, with dashed red lines showing 95% confidence intervals derived from restricted cubic spline regressions. Reference lines for no association are indicated by the grey bold lines at an odds ratio of 1.0. Vertical blue bars represent the frequency of ICM volume in a histogram. ICM, iodinated contrast media, OR, odds ratio, CI, confidence interval.

Among all patients, the distribution ICM volume was left-skewed, ranging from 80 to 530 mL with 25th, 50th and 75th percentile being 120, 150 and 220mL, respectively. To illustrate the association between ICM volume and clinical outcomes, we modeled ICM volume as RCS to provide a smooth and flexible description of their linear dose-response relationship. As displayed in **Figure 2**, ICM volume is associated with the risk of the composite endpoint (*P* value for non-linear spline terms < 0.0001), hyperthyroidism (*P* < 0.0001), overt hyperthyroidism (*P* < 0.0044) and subclinical hyperthyroidism (*P* < 0.0028) with a non-linear dose–response relationship (**Figure 2**).

From restricted cubic splines, a cut-off value of 189.23ml was derived for composite endpoint (*OR* = 1), 205.11ml for hyperthyroidism (*OR* = 1), 211.34ml for overt hyperthyroidism (*OR* = 1) and 193.28ml for subclinical hyperthyroidism (*OR* = 1). To provide an ICM volume value for clinical outcomes, a threshold of 200ml was determined according to the combined consideration of RCS results (**Figure 2**). Base on it, the included patients were divided into low-volume group (ICM volume <= 200ml) and high-volume group (ICM volume > 200ml).

The analysis population included 1381 patients in the low-volume group and 681 patients in the high-volume group. Between the two groups, the baseline demographic characteristics, medical history, diagnosis, medications and thyroid function were comparable. Compared to low-volume group, patients in high-volume group receive more stents (1.29 ± 0.50 vs. 1.59 ± 0.77, *P* < 0.0001). And the complexity of the PCI procedure has a significant difference between the two groups (109 ± 7.9 vs. 423 ± 62.1, *P* < 0.0001) (**Table 1**).

**Table 1:** Characteristics of the patients at baseline.

Variables	Low-volume group (n=1381)	High-volume group (n=681)	*P* value
**Demographic characteristics**
Age (years)	62.99 ± 9.76	61.45 ± 9.75	0.993
Male—no. (%)	1110 (80.4)	559 (82.1)	0.353
BMI (Kg/m^2^) ^*^	23.94 ± 3.11	23.90 ± 3.04	0.477
Serum Creatinine (umol/L)	79.24 ± 20.72	80.43 ± 20.46	0.499
**Behavioral habit**			
Current smoker—no. (%)	592 (42.9)	282 (41.4)	0.529
**Medical History**			
Hypertension—no. (%)	45 (3.3)	17 (2.5)	0.341
Diabetes mellitus—no. (%)	429(31.1)	218(32.0)	0.663
Hyperlipidemia —no. (%)	434 (31.4%)	222 (32.6)	0.591
Peripheral vascular disease—no. (%)	498 (36.1)	232 (34.1)	0.373
Prior stroke—no. (%)	136(9.8)	63(9.3)	0.666
Clinical Diagnosis			0.938
Unstable angina pectoris—no. (%)	744 (53.9)	363 (53.3)	
Stable angina pectoris—no. (%)	268 (19.4)	131 (19.2)	
NSTEMI—no. (%)	369 (26.7)	187 (27.5)	
**PCI procedure characteristics**			
Number of implanted stents	1.29 ± 0.50	1.59 ± 0.77	< 0.0001^†^
I CM Volume (ml)	120(120.150)	220 (250.320)	< 0.0001^†^
Complex PCI—no. (%)	109 (7.9)	423 (62.1)	< 0.0001^†^
**Medications at baseline**			
Aspirin—no. (%)	1335 (96.7)	664 (97.5)	0.300
Clopidogrel or Ticagrelor—no. (%)	1334 (96.6)	661 (97.1)	0.574
Statins —no. (%)	1281 (99.1)	673 (98.8)	0.620
**Thyroid function**			
TSH (MIU/L)	1.733 ± 1.061	1.759 ± 1.024	0.204
FT3 (pmol/L)	4.657 ± 0.692	4.689 ± 0.640	0.308
FT4 (pmol/L)	11.84 ± 2.11	11.79 ± 2.15	0.623

Values are described as mean ± SD, median (quartile1, quartile3) or number (percentage).

Counting variables were compared using chi-squared test. Normally distributed variables were compared using independent sample t test, and non-normally distributed variables using Mann-Whitney test.

*Calculated as weight (kilograms) divided by squared height (meters).

^†^P < 0.05

BMI, body mass index; NSTEMI, non-ST elevation myocardial infarction; ICM, iodinated contrast media.

During the study period, 33 patients of the low-volume group had events——1 overt hyperthyroidism, 13 subclinical hyperthyroidism, 8 overt hypothyroidism, and 11 subclinical hypothyroidism, while 28 patients of the high-volume group had events——5 overt hyperthyroidism, 11 subclinical hyperthyroidism, 8 overt hypothyroidism, and 4 subclinical hypothyroidism.

### Changes in Thyroid Function

When comparing thyroid function between groups, with baseline values as a covariable, there was a significant increase in serum FT4 at the one-year PCI intervention. Specifically, the high-volume group was 0.238 ± 0.092 pmol/L higher than the low-volume group (*P* = 0.010) in the serum FT4. In contrast, no significant differences were observed between groups in serum TSH (95% *CI*, -0.307-0.001, *P* = 0.048) and serum FT3 (95% *CI*, -0.030-0.085, *P* = 0.348) after one year (**Table 2**).

**Table 2:** Comparison of thyroid function between the low-volume group and high-volume group at the 12th month.

Variables	Low-volume group(change from baseline)	High-volume group(change from baseline)	Difference inMeans ± *SE*	*P* values(Low-volume group vs.High-volume group)	95% *CI*
TSH (MIU/L)	1.823 ± 0.045	1.669 ± 0.064	-0.154 ± 0.078	0.048^†^	-0.307, 0.001
FT3 (pmol/L)	4.787 ± 0.017	4.814 ± 0.024	0.027 ± 0.029	0.348	-0.030, 0.085
FT4 (pmol/L)	12.040 ± 0.053	12.278 ± 0.076	0.238 ± 0.092	0.010^†^	0.057, 0.419

Values are absolute differences in arithmetic means ± SE. The 12^th^-month P values and 95% CI were derived from ANCOVA with adjustment for the baseline values.

^†^P < 0.05.

SE, standard error; CI, confidence interval; TSH, thyroid stimulating hormone; FT3, free triiodothyronine; FT4, free thyroxine.

### Logistic Analysis for Endpoints

Calibration for all models was adequate with non-significant statistical result (**eTable 1**). The binary regression analysis results are shown in **Table 3**. In the crude model, there was a significant difference in composite between the two groups. (*OR* 1.75, 95% *CI* (1.05, 2.92), *P* = 0.032). After adjusted confounding factors, the same result was demonstrated in model1 and model2(*OR* 1.73, 95% *CI* (1.01-2.96), *P* = 0.032 and *OR* 1.83, 95% *CI* (1.09-3.06), *P* = 0.022, respectively). The results also showed a positive correlation for hyperthyroidism in all models (*OR* 2.35, 95% *CI* (1.14-4.84), *P* = 0.021, *OR* 10.36, 95% *CI* (1.20-89.00), *P* = 0.033 and *OR* 2.35, 95% *CI* (1.13-4.87), *P* = 0.022, respectively). Of note, similar observations were acquired in crude model and adjusted model II when and overt hyperthyroidism as the endpoint (*OR* 10.21, 95% *CI* (1.19, 87.54), *P* = 0.034 and *OR* 10.73, 95% *CI* (1.24-92.72), *P* = 0.031, respectively). Yet null results were found in subclinical hyperthyroidism, hypothyroidism, overt hypothyroidism and subclinical hypothyroidism.

**Table 3:** Logistic regression analysis for after 1-year clinical outcomes.

Events	Low-volume group(n=1381) *no. of patients (%)*	High-volume group(n=681) *no. of patients (%)*	Crude model		Model1		Model2	
			*OR* (95%*CI*)	*P* value	*OR* (95%*CI*)	*P* value	*OR* (95%*CI*)	*P* value
**Composite endpoint**	33 (2.4)	28 (4.1)	1.75 (1.05-2.92)	0.032^†^	1.73 (1.01-2.96)	0.045^†^	1.83 (1.09-3.06)	0.022^†^
Overt hyperthyroidism or overt hypothyroidism or subclinical hyperthyroidism or subclinical hypothyroidism								
**Hyperthyroidism**	14 (1.0)	16 (2.3)	2.35 (1.14-4.84)	0.021^†^	10.36 (1.20-89.00)	0.033^†^	2.35 (1.13-4.87)	0.022^†^
Overt hyperthyroidism	1 (0.1)	5 (0.7)	10.21 (1.19-87.54)	0.034^†^	1.78 (0.79-4.02)	0.164	10.73 (1.24-92.72)	0.031
Subclinical hyperthyroidism	13 (0.9)	11 (1.6)	1.73 (0.77-3.88)	0.185	1.84 (0.80-4.25)	0.153	1.68 (0.74-3.81)	0.215
**Hypothyroidism**	19 (1.4)	12 (1.8)	1.29 (0.62-2.66)	0.498	1.36 (0.65-2.82)	0.411	1.36 (0.65-2.83)	0.413
Overt hypothyroidism	8 (0.6)	8 (1.2)	2.04 (0.76-5.46)	0.156	2.21 (0.82-5.93)	0.118	2.21 (0.82-5.96)	0.118
Subclinical hypothyroidism	11 (0.8)	4 (0.6)	0.74 (0.23-2.32)	0.601	0.76 (0.24-2.40)	0.641	0.75 (0.24-2.39)	0.629

Binary logistic regression analysis with enter method was performed to determine the association between noncomplex PCI group and complex PCI group for thyroid disease.

Crude model: Unadjusted model.

Model 1, adjusted for age, gender and obesity.

Model 2, adjusted for age, gender, obesity, current smoker, hypertension, hyperlipemia and previous stroke.

^†^P < 0.05.

OR, odds ratio; CI, confidence interval.

### Analysis for PCI Complexity

The median (quartile1, quartile3) of ICM volume for non-complex PCI patients were 50% higher than non-complex PCI patients (250 vs.120 mL, *P* < 0.0001). (eFigure). Specifically, the serum TSH of complex PCI patients was 0.210 ± 0.084 (95% *CI*, 0.046-0.374, *P* = 0.012) MIU/L higher, and the serum FT4 was 0.242 ± 0.099 (95% *CI*, 0.047-0.437, *P* = 0.015) pmol/L higher than non-complex PCI patients (**eTable 2**).

In the crude model, there was no significant difference for outcome between the non-complex PCI group and the complex PCI group. However, in adjusted model I and adjusted model II, patients received complexed PCI had an increased risk of composite endpoint (*OR* 1.73, 95% *CI* (1.01-2.96), *P* = 0.045 and *OR* 1.86, 95% *CI* (1.06-3.28), *P* = 0.032, respectively). Of note, a positive correlation was acquired in adjusted model II when hyperthyroidism was endpoint (*OR* 2.28, 95% *CI* (1.03, 5.01), *P* = 0.041). Yet null results were found in hypothyroidism. (**Table 4**).

**Table 4:** Subgroup analysis based on non-complex PCI and complex PCI population for 1-year clinical outcomes.

	Non-complex PCI (n=1530)*no. of patients (%)*	complex PCI (n=532)*no. of patients (%)*	Crude model		Model1		Model2	
Events			*OR* (95%*CI*)	*P* value	*OR* (95%*CI*)	*P* value	*OR* (95%*CI*)	*P* value
**Composite end point**	39 (2.5)	22 (4.1)	1.65 (0.97-2.81)	0.065	1.73 (1.01-2.96)	0.045^†^	1.86 (1.06-3.28)	0.032^†^
Overt hyperthyroidism or overt hypothyroidism or subclinical hyperthyroidism or subclinical hypothyroidism								
**Hyperthyroidism**	18 (1.3)	12 (2.3)	1.94 (0.93-4.05)	0.078	1.99 (0.95-4.17)	0.068	2.28 (1.03-5.01)	0.041^†^
Overt hyperthyroidism	3 (0.2)	3 (0.6)	2.89 (0.58-14.35)	0.195	2.66 (0.53-13.24)	0.233	3.68 (0.71-19.13)	0.122
Subclinical hyperthyroidism	15 (1.0)	9 (1.7)	1.74 (0.76-4.00)	0.193	1.84 (0.80-4.25)	0.153	1.79 (0.73-4.40)	0.204
**Hypothyroidism**	21 (1.6)	10 (1.9)	1.38 (0.64-2.94)	0.410	1.46 (0.68-3.15)	0.327	2.03 (0.73-5.64)	0.177
Overt hypothyroidism	9 (0.6)	7 (1.3)	2.25 (0.84-6.08)	0.109	2.34 (0.86-6.33)	0.095	2.80 (0.55-14.2)	0.214
Subclinical hypothyroidism	12 (0.8)	3 (0.6)	0.72 (0.20-2.55)	0.608	0.78 (0.22-2.80)	0.706	0.80 (0.21-3.28)	0.736

Binary logistic regression analysis with enter method was performed to determine the association between noncomplex PCI group and complex PCI group for thyroid disease.

Crude model: Unadjusted model.

Model 1, adjusted for age, gender and obesity.

Model 2, adjusted for age, gender, obesity, current smoker, hypertension, hyperlipemia and previous stroke.

^†^P < 0.05.

PCI, percutaneous coronary intervention; OR, odds ratio; CI, confidence interval.

## Discussion

This is the largest retrospective study performed to date to assess the impact of ICM exposure on thyroid disease for patients received PCI. First, we demonstrated that after 1-year PCI, the serum level of FT4 was significantly increased. Second, the volume of ICM correlated with composite endpoint and hyperthyroidism with a nonlinear dose-response relationship. Third, the risk of hyperthyroidism increased in high-volume ICM group patients.

ICM is routinely used in coronary angiography and PCI procedures. The general procedure might use an average 200ml dose of ICM containing 35µg/ml provides 7,000 µg free iodide, far higher than the recommended daily allowance of 150μg for adults (22). Sudden exposure to high iodine levels may cause thyroid dysfunction, especially in patients with nodular goiter and thyroid dysfunction (6, 23, 24). But currently, for the general population, there are no guidelines for routinely screening thyroid function before and after PCI (25). Monitoring for thyroid dysfunction after ICM exposure is only recommended in high-risk patients (26). However, the last decade has witnessed a significant shift in characteristics of the CAD patient population received PCI, including an increase in mean age and prevalence of diabetes, hypercholesterolemia, hypertension and chronic kidney disease, among others. Consequently, the frequency of myocardial infarction and the amount of PCI procedures also increased. Due to the patient-related and angiographic factors, the overall quantity and complexity of PCI have increased (1). That gives a result to a higher dose of ICM patients suffered. But the association between ICM volume and long-term risk of thyroid disease and necessity of thyroid function monitoring still unclear.

The observed association between ICM exposure and hyperthyroidism is likely explained by the iodine or iodide load conveyed by ICM. Iodine is actively transported into the thyroid gland by the Na+/I- symporter(NIS) (27). NIS gene expression and membrane localization are stimulated by TSH (28). Once in thyroid follicles, iodine is used for the synthesis of the thyroid hormones, T4 and T3. That could explain why serum level of FT4 has a significant difference before and after PCI in our study. The mechanism of ICM-induced hyperthyroidism involves impaired autoregulation following an iodine load. The occurrence of thyrotoxicosis arising from excess iodine exposure is termed the Jöd-Basedow phenomenon (9). Excess iodine intake will result in transient or permanent hyperthyroidism (29, 30). The association between hyperthyroidism and ICM-exposure in our study may be explained based on this mechanism. Still, in certain study, thyroid dysfunction of ICM exposure occurred especially in patients had a history of thyroid disease, or living in an iodine-deficient area, or had renal insufficiency (4, 29, 31). And patients with certain prescriptions, such as amiodarone, lithium, and α-interferon, were also considered having more risk of thyroid dysfunction (32). But our study was based on thyroid normal patients. We speculated that the increased risk of hyperthyroidism may due to much higher dose of ICM explored in complex PCI procedure.

With respect to the mechanism of ICM-induced hypothyroidism, the disorder probably occurs when the thyroid gland fails to adapt to the acute Wolff-Chaikoff effect after excess iodine intake. But the symptoms are usually mild and transient, and the vulnerable populations tend to have Hashimoto’s thyroiditis, previously treated Graves’ disease, thyroid operation, and postpartum thyroiditis (8). The observative population was patients with euthyroidism. That may explain the null result in hypothyroidism by above mechanism.

Several studies indicated that ICM exposure can cause thyroid disease (4–7, 23, 33, 34). Rhee, C. M.et al performed a nested case-control study in the euthyroid population. They demonstrated that ICM exposure was a significant associate with hyperthyroidism and overt hypothyroidism. But the baseline data lacked contrast volume and osmolarity (23). A prospective observational cohort study in 2013 investigated long-term effects for thyroid function in patients with euthyroidism. undergoing coronary angiography using nonionic ICM. They found out that ICM may cause subclinical hyperthyroidism after 8 weeks (5). In Taiwan, a retrospective cohort study randomly selected 1 million people, recruiting 19 642 ICM-exposure cases and 78 568 matched non-ICM-exposure controls. After 6-year observation, patients with ICM exposure had a significantly higher risk of thyroid dysfunction [HR, 1.46, 95% CI (1.29-1.66)]. The same result was confirmed in hyperthyroidism [HR, 1.22, 95% CI (1.04-1.44)] and hypothyroidism [HR, 2.00, 95% CI (1.65-2.44)] (6). Bonelli, N.et al. performed a cross-sectional study in 2018. After 1 year follow up for 810 consecutive ischemic heart disease patients with unknown thyroid diseases or treatment with thyroid-related drugs received coronary angiography. It is investigated that the prevalence of spontaneous subclinical hyperthyroidism in ischemic heart disease is surprisingly elevated and is further increased by iodine load, particularly in patients with thyroid nodules and familial history of thyroid diseases, persisting in a not negligible number of them even after one year (7).

Compared to the above studies, the strengths are obvious in our study. First, the sample size is larger than previous studies, which improved the statistical power. Second, patients in our study are strictly chosen from an iodine-sufficient area in the southeast coastal region of China. And our study was carefully designed to record exact dose and detailed ICM osmolarity. Those were not considered in above study. Third, dose-response relationships between ICM volume and composite end point and hyperthyroidism were explored using restricted cubic splines, which made the associations more visual and clearer. Finally, we conducted further analysis based on complexity of PCI procedure. The positive result of that is of great importance to the daily clinical practice of PCI. However, limitations do exist in our study. First, our study was limited by its retrospective nature. Second, patients in this study were predominantly men. Therefore, the results may not be extrapolated to women. Third, it is fairly clear that radiation may associated with a risk of thyroid disorder (35). But due to the lack of data on exposure duration, we cannot adjust the bias of radiation-related outcome. That may lead to deviation for endpoints. Fourth, iodine status is a key determinant of thyroid disorders in adults. Severe iodine deficiency causes goiter and hypothyroidism (36). Though the patients in our study are reside in coastal region, an iodine-sufficient area, the difference in individual diet may lead to bias in our conclusion. Fifth, the laboratory measurements for 1, 3, 6 months was sometimes conducted in community hospitals and the data were inadequate. Unfortunately, further exploration that at which month when thyroid function and thyroid disease began to show significant differences cannot be performed. Finally, due to the difficulty in diagnosis for asymptomatic autoimmune disease and goiter, we may mistakenly include this population. That may lead to bias for clinical outcomes.

## Conclusion

The present analysis gives an overview that ICM volume is associated with an increased risk of thyroid dysfunction and thyroid disease. Given the pervasive use of ICM in contemporary practice and the known sequelae of thyroid functional disorders, regular examinations of thyroid function before and after PCI are needed, especially for patients received high-volume ICM. Physicians and patients should be aware of the potential thyroidal complications associated with ICM exposure and novel effective strategies should be investigated to decrease the risk of this complication.

## Data Availability Statement

The original contributions presented in the study are included in the article/**Supplementary Material**. Further inquiries can be directed to the corresponding authors.

## Ethics Statement

The studies involving human participants were reviewed and approved by Fujian Medical University Union Hospital. The ethics committee waived the requirement of written informed consent for participation.

## Author Contributions

YC contributed to data analysis and editing the manuscript. XZ and NL contributed to data analysis and conceptualization. WN and BH contributed revising the manuscript. XY contributed to data collection and conceptualization. CL and YL contributed to project administration and supervision. All authors contributed to the article and approved the submitted version.

## Funding

This study was supported by the National Natural Science Foundation of China grants (82120108004) to CL.

## Conflict of Interest

The authors declare that the research was conducted in the absence of any commercial or financial relationships that could be construed as a potential conflict of interest.

## Publisher’s Note

All claims expressed in this article are solely those of the authors and do not necessarily represent those of their affiliated organizations, or those of the publisher, the editors and the reviewers. Any product that may be evaluated in this article, or claim that may be made by its manufacturer, is not guaranteed or endorsed by the publisher.
