# Supplementary figures and images for: Impact of Iodinated Contrast Media in Patients Received Percutaneous Coronary Intervention: Focus on Thyroid Disease
Source: Front Endocrinol (Lausanne). 2022 Jun 23;13:917498. doi: 10.3389/fendo.2022.917498 (PMC9259844; doi:10.3389/fendo.2022.917498)

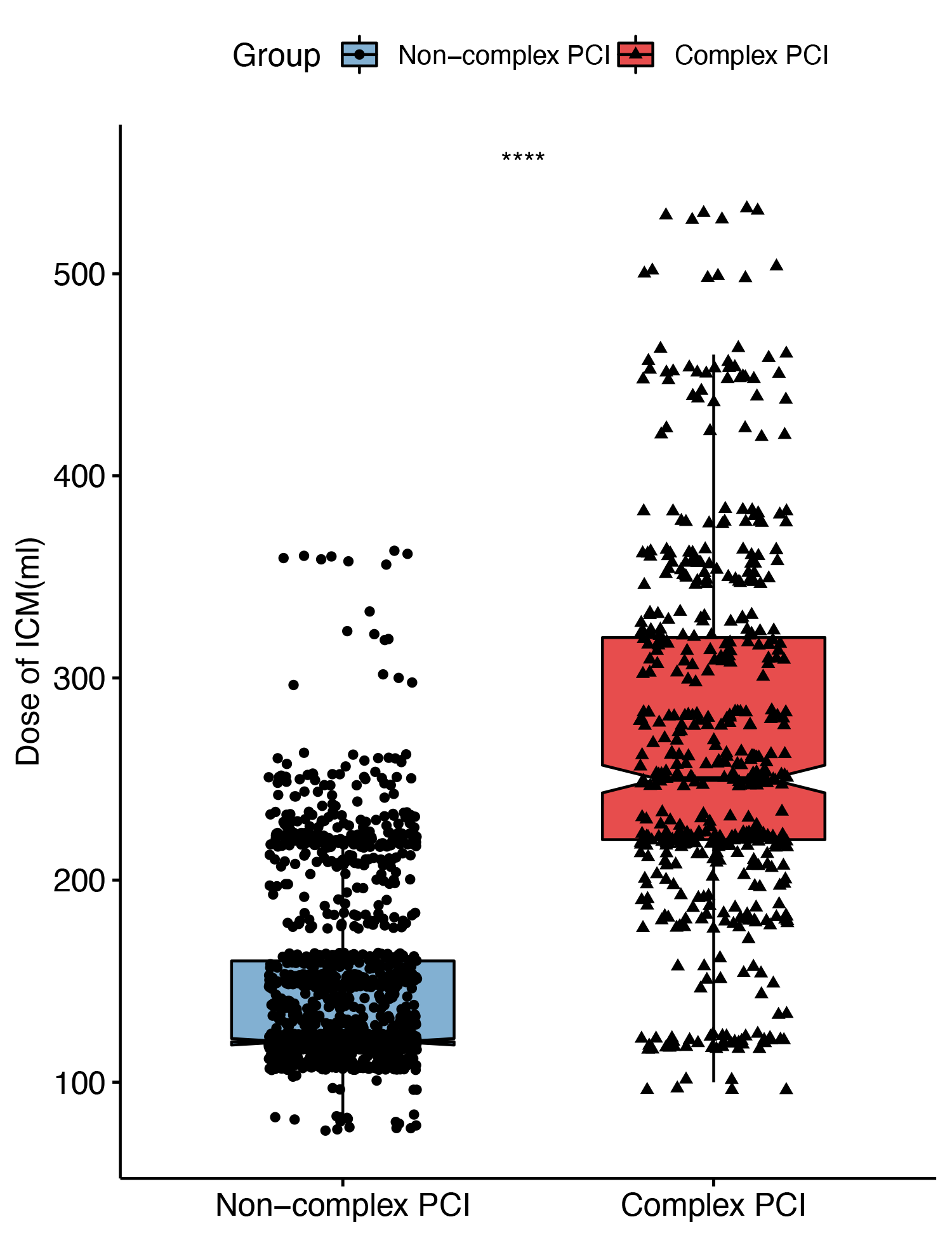

Supplement: Supplementary file 1 [file Image_1.tif]
