# Supplementary material for: Impact of Iodinated Contrast Media in Patients Received Percutaneous Coronary Intervention: Focus on Thyroid Disease
Source: Front Endocrinol (Lausanne). 2022 Jun 23;13:917498. doi: 10.3389/fendo.2022.917498 (PMC9259844; doi:10.3389/fendo.2022.917498)
Supplement: Supplementary file 3 [file DataSheet_2.docx]

**Supplementary Materials**

**eTable1.** **The fit goodness of logistic regression models for clinical outcomes between low-volume group and high-volume group.**

**eTable2. Comparison of thyroid function between noncomplex PCI group versus complex PCI group at the 12^th^ month.**

**R code for restricted cubic spline**

**eFigure.** **Comparison of dose of ICM between complex PCI group and non-complex PCI group.**

Each patient is represented by a single point or triangle. The lines within boxes represent medians, while the edges of the boxes represent lower and upper quartiles. The whiskers represent the range of the data set.

**P* < 0.05, ***P* < 0.01, ****P* < 0.001, *****P* < 0.0001.

Abbreviation: ICM, iodinated contrast media; PCI, percutaneous coronary intervention.

**eTable1. Hosmer-Lemeshow Test for clinical outcomes between low-volume group and high-volume group.**

| **Events** | **Low-volume group  (n=1381)**  ***no. of patients (%)*** | **High-volume group  (n=681)**  ***no. of patients (%)*** | **Model1** | | **Model2** | |
| --- | --- | --- | --- | --- | --- | --- |
|  |  |  | **χ2-value** | **P value** | **χ2-value** | **P value** |
| **Composite endpoint** | 33 (2.4) | 28 (4.1) | 1.782 | 0.776 | 5.227 | 0.733 |
| Overt hyperthyroidism or  overt hypothyroidism or  subclinical hyperthyroidism or  subclinical hypothyroidism |  |  |  |  |  |  |
| **Hyperthyroidism** | 14 (1.0) | 16 (2.3) | 3.087 | 0.543 | 6.266 | 0.618 |
| Overt hyperthyroidism | 1 (0.1) | 5 (0.7) | 1.940 | 0.857 | 6.846 | 0.553 |
| Subclinical hyperthyroidism | 13 (0.9) | 11 (1.6) | 2.401 | 0.662 | 10.02 | 0.264 |
| **Hypothyroidism** | 19 (1.4) | 12 (1.8) | 0.801 | 0.938 | 11.772 | 0.162 |
| Overt hypothyroidism | 8 (0.6) | 8 (1.2) | 0.966 | 0.915 | 8.724 | 0.366 |
| Subclinical hypothyroidism | 11 (0.8) | 4 (0.6) | 2.004 | 0.849 | 10.486 | 0.233 |

Chi-Square values and P values and 95%CI were derived from Hosmer-Lemeshow test.

**eTable2. Comparison of thyroid function between noncomplex PCI group versus complex PCI group at the 12^th^ month.**

| **Variables** | **Non-complex PCI  (change from baseline)** | **Complex PCI  (change from baseline)** | **Difference in  Means ± SE** | **P values  (complex PCI vs  non-complex PCI)** | **95% CI** |
| --- | --- | --- | --- | --- | --- |
| TSH (MIU/L) | 1.765±0.042 | 1.975±0.072 | 0.210±0.084 | **0.012** | 0.046, 0.374 |
| FT3 (pmol/L) | 4.795±0.016 | 4.799±0.027 | 0.005±0.031 | 0.884 | -0.057, 0.066 |
| FT4 (pmol/L) | 12.022±0.050 | 12.265±0.086 | 0.242±0.099 | **0.015** | 0.047, 0.437 |

Values are absolute differences in arithmetic means ± SE. The 12^th^-month P values and 95%CI were derived from ANCOVA with adjustment for the baseline values.

Abbreviations: SE, standard error; CI, confidence interval; PCI, percutaneous coronary intervention; TSH, thyroid stimulating hormone; FT3, free triiodothyronine; FT4, free thyroxine.

**R code for restricted cubic spline**

library(rms)

library(survival)

data = read.csv("/Users/chenyasha/Documents/ICM and thyrorid function/frontiers/R.csv",header= TRUE)

dd <- datadist(data)

options(datadist='dd')

fit <- lrm(clinicalhyper ~ CMV,data=data)

fit=update(fit)

anova(fit)

OR<-Predict(fit, CMV,fun=exp,ref.zero = TRUE)

par(mar=c(3,4,1,5))

col=c("#CCEDFC", "#f75f6c","#6F6F6F")

hist(data$CMV,

axes=F,

xlab="",ylab="",

xlim=c(80,530),

col=col[1],

border="white",

breaks=20,

main='',

freq=T)

axis(4, las = 1)

par(new=T)

plot(OR[,1],axes=T,OR$yhat,type='l',lty=1,lwd=2,main = "Overt hyperthyroidism",

col=col[2],

xlim = c(80,530),

ylim=c(0,20),las = 1,

cex.axis = 1,

xlab='Volume of ICM (ml)',ylab='OR (95%CI)')

axis(2, las = 1, at = 1, labels = "1")

lines(OR[,1],OR$lower,type='l',lty=2,lwd=2,col=col[2])

lines(OR[,1],OR$upper,type='l',lty=2,lwd=2,col=col[2])

abline(h = 1,lwd=2,col = "#6F6F6F")

mtext("Frequency",side = 4,line = 3.5,las = 3, font = 1,cex = 1.15, las = 0)
